# Diwaniyyah, dietary habits and daily activity during Ramadan among Kuwaiti Men

**DOI:** 10.1017/S1368980026102079

**Published:** 2026-02-11

**Authors:** Maha Meshari Al-Sejari, Jasem Bader Al-Failakawi, Faisal Khuzaim Al-Khuzaim, Yagoub Yousif Al-Kandari

**Affiliations:** College of Social Sciences, Kuwait University, Kuwait City, Kuwait

**Keywords:** Diwaniyyah, Dietary habits, Daily activity, Ramadan, Men’s health, Kuwait

## Abstract

**Objective:**

The major aim was to examine the interaction between visiting Kuwaiti *Diwaniyyah*, dietary habits and daily activities during Ramadan among Kuwaiti men.

**Design:**

An electronic questionnaire was used to collect data. The questionnaire included questions about various sociocultural factors, frequency of visiting *Diwaniyyah*, dietary habits and physical activity variables. For the sociocultural variables, age, level of education and governorate were collected. ANOVA, *t* test, Pearson’s correlation and regression were used.

**Setting:**

Respondents came from different and various subgroups of the Kuwaiti population and from all six governorate

**Participants:**

A total of 736 Kuwaiti men were selected from an opportunistic sample.

**Results:**

The results show that the food most eaten at night after Iftar in general was rice, meat and dairy products. Younger age groups eat more sweets, pastry, traditional sweets, dairy products and rice than the other age groups. The higher the frequency of visiting *Diwaniyyah*, the greater the consumption of these types of food at night. Also, older men eat fewer types of food and have lower physical activity levels at night during Ramadan than other age groups. Men with graduate educational levels who regularly visit *Diwaniyyah* consume more types of food at night than those who do not visit the *Diwaniyyah*. Age, social individuals and number of days visiting *Diwaniyyah* were associated with and predicted the number of types of food eaten at night during Ramadan.

**Conclusion:**

The study findings reveal that during Ramadan, lifestyle behaviour changes among men, including the timing and number of meals, type and portion of food consumption and physical activity behaviour.

A fundamental part of Kuwaiti culture is *Diwaniyyah*, which refers to a place where men socially gather and discuss local and national issues related to policy, education, health, society and the economy^(1,2)^. Usually, *Diwaniyyah* are located in residential areas attached to private houses and have regular members who attend it daily or weekly depending on the host’s schedule and resources. In addition to the main function of *Diwaniyyah* as a source of social interaction and information exchange, attendees share meals, play traditional games and freely express their thoughts and opinions about any topic^(1)^.

Owing to its significant impact on Kuwaitis’ daily life schedules, physical activities, dietary habits and mental health, many studies of *Diwaniyyah* in Kuwait have been conducted from different academic perspectives^(3–6)^. The outcomes of previous studies have confirmed the essential role of attending *Diwaniyyah* in Kuwaiti families. The current study aimed to investigate the lifestyle behaviours of Kuwaiti men attending *Diwaniyyah* during Ramadan.

Ramadan is the ninth month of the Islamic lunar calendar. During Ramadan, Muslims fast daily from dawn until sunset, a fasting period when it is prohibited to partake of food and drink^(7)^. Culture has a significant influence on food choices, especially during special occasions such as Ramadan^(8)^. In Kuwait, food options change drastically and some food items are consumed only during Ramadan^(9)^, such as *sambosa* (deep-fried filled triangular pasties), *tashreeb* (broth-soaked bread topped with meat and/or vegetables), *harees* (made with wheat grain, meat and ghee) and *qatayef* (sweet folded pancake filled with cream or nuts)^(9)^. All these dishes are high in carbohydrates, fat and energies. Therefore, the energies consumed daily during Ramadan differ from those consumed during other months^(10)^.

Daily physical activity patterns during Ramadan also change as a result of fasting among men who attend *Diwaniyyah.* Previous studies^(11–14)^ have shown that physical activity levels often decrease – or remain unchanged – compared to pre-Ramadan periods. This reduction is linked to several factors: most physical activity typically occurs during daylight hours when the body is not in its optimal state for exertion, and many Muslims engage in Taraweeh prayers after Iftar, limiting time and energy for exercise. Research also indicates that fasters often adopt more sedentary behaviours during the day, such as watching television or sleeping for longer periods^(15–19)^.

The significance of the current study lies in its focus on providing a descriptive comparative analysis of daily activities and dietary habits among Kuwaiti men during Ramadan, particularly those who attend the *Diwaniyyah*. While previous studies have examined energy and macronutrient intake during Ramadan in general^(11,15,20–31)^, none have explored how these nutritional patterns are shaped within the cultural and social context of the *Diwaniyyah*. By adopting an anthropological perspective, this study goes beyond measuring dietary intake and situates eating behaviours within a central Kuwaiti cultural institution. This approach offers a unique contribution by linking nutritional practices and physical activity to cultural traditions, thereby providing a deeper understanding of how social environments influence men’s routines and food consumption during Ramadan.

The major objective of this study was to examine the interaction between visiting Kuwaiti *Diwaniyyah*, dietary habits and daily activities during Ramadan among Kuwaiti men.

This study aimed to answer the following research questions:

1. What is the relationship between demographic characteristics, *Diwaniyyah* attendance patterns and dietary habit and physical activity scores among regular and non-regular *Diwaniyyah* attendees?
2. Which factors predict dietary habits and physical activity level?

## Ecological model of food and nutrition

This study aimed to use an ecological model of food and nutrition to enhance the understanding of dietary changes among Kuwaiti men who visit *Diwaniyyah*. The strength of this model lies in the reciprocal relationship it posits among various environmental factors (social environment, technology, physical environment, social organisation and culture), where the position of each factor can affect the nutritional requirements and status of specific populations. This feature makes the model dynamic and interactive, allowing modifications in one sector to trigger changes in other parts of the model, showcasing the interconnectedness of the elements^(32)^. Furthermore, the incorporation of global forces enhances the model’s biocultural perspective, making it an excellent framework for analysing changes in diet, diet quality and energy balance within modern populations^(33)^.

**Figure 1:**
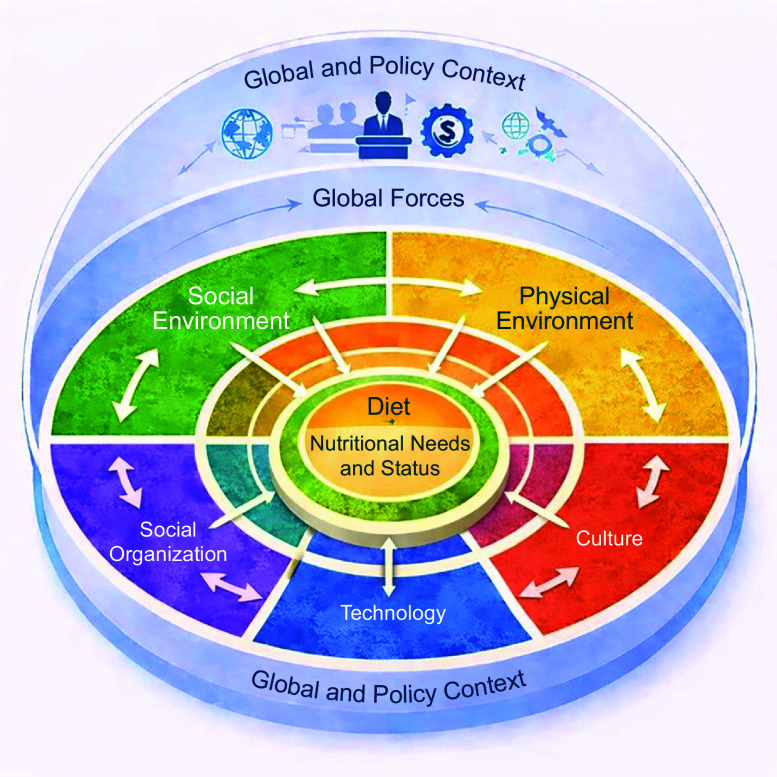
Ecology model.

The ecological model emphasises that health behaviours, such as food choices and physical activity, are shaped by multiple interacting levels of influence (see Figure 1). In this study, we apply the model by focusing specifically on two key levels: the cultural and the social–environmental influences. At the cultural level, Ramadan represents a powerful set of religious and social norms that restructure daily life. Fasting during daylight hours alters eating schedules, influences the types of foods traditionally consumed during Iftar and Suhoor and may modify patterns of physical activity. Cultural expectations during Ramadan often promote communal eating, increased evening socialisation and specific culinary traditions that may encourage higher-energy or carbohydrate-rich foods. In addition, the spiritual and social rhythm of Ramadan – such as late-night prayers (Taraweeh) and altered sleep patterns – further affects when individuals choose to rest, exercise, or engage in sedentary behaviours.

At the social–environmental level, the *Diwaniyyah* serves as a central social institution that shapes men’s daily routines and behaviours. It influences dietary choices through the types of foods commonly served, the social pressure to eat with peers and the hospitality norms that encourage sampling multiple dishes. The *Diwaniyyah* may also affect physical activity patterns by providing an evening social space that can replace time otherwise spent in active pursuits. For many attendees, visiting the *Diwaniyyah* becomes a routine that structures their evening and night-time behaviours, potentially reinforcing more sedentary lifestyles during Ramadan.

By examining both the cultural context of Ramadan and the social dynamics of the *Diwaniyyah*, this study applies the ecological model to highlight how broader cultural norms and immediate social environments interact to shape men’s dietary habits and physical activity. This approach allows for a more comprehensive understanding of health-related behaviours by recognising that individual choices are embedded within, and influenced by, the cultural and social structures surrounding them.

## Methods

### Sample

A total of 736 respondents were selected from an opportunistic sample. Participants were approached in public and social settings, and those who met the study criteria were invited to participate. Inclusion criteria were being Kuwaiti, Muslim men who were fasting during Ramadan. Individuals who were non-Kuwait, non-Muslim, female or not fasting were excluded from the study. Participation was voluntary in this study. Three months of data were gathered immediately following Ramadan in 2023, and all findings were presented prior to December 2023. This research is a part of a large project that conducted in Kuwait by the researcher. The men voluntarily responded to the questionnaire. Informed consent was obtained from all participants, and all study procedures were conducted in accordance with the ethical guidelines and regulations of College of Social Sciences at Kuwait University.

### Variables

An electronic questionnaire was used to collect data. The questionnaire included questions about various sociocultural factors, frequency of visiting *Diwaniyyah*, dietary habits and physical activity variables. For the sociocultural variables, information about the respondents’ age, level of education and governorate was collected. Age was divided into four categories: 20 years or below, 21–40 years, 41–59 years and 60 years or above. A single-item self-rating scale of the participants’ economic status (from *very low* = 1 to *very high* = 10) was used to measure their socioeconomic status. Eight educational levels were identified and divided into three categories: low (high school or below), medium (university level) and high (higher education). Questions about the number of days visiting *Diwaniyyah* weekly and the average hours spent in *Diwaniyyah* daily on weekdays and weekends during the first 20 d of Ramadan were included; the last 10 d were excluded because many Kuwaiti men go to the mosque then for late-night prayers *(Qeyam)*. The number of days visiting *Diwaniyyah* was divided into four categories: 6–7 d, 3–5 d, 1–2 d and less than 1 d or never visited. For comparison purposes, it also divided participants into those regularly visiting *Diwaniyyah* (6–7 d, 3–5 d and 1–2 d) and those not visiting it. A single-item self-rating scale of social individuals (people who are social) (from *very low* = 1 to *very high* = 6) was used to measure respondents’ social networking.

Two scales were used in this study:

1. Dietary habit scale: This scale was developed by the first and fourth researchers. It includes fourteen items for different types of food. These foods were chosen based on two criteria: first, they fall into one of the six food pyramid divisions; second, they are among the most popular foods in the local culture. This scale was developed after reviewing the food pyramid, and it includes meat, chicken, seafood, pastry, vegetables, dairy products, fruits, whole grains, rice, sweet and traditional sweets. It also includes the ways or types of cooking, such as barbeque, frying and juice. After examining the sections of the gendered food pyramid – grains, vegetables, fruits, protein foods, dairy, fats and sweets – the dietary habit scale was created. Researchers have selected these foods because they are the most popular in Kuwaiti culture. Five Kuwait University academic members examined and validated various kinds of food. The respondents were asked to determine the degree to which they ate these types of food at night after *Iftar* during the first 20 d of Ramadan. In addition, the respondents were asked to determine the extent to which they eat dinner late at night (*Qabka*), scored on a six-point Likert scale, ranging from *very much* (6) to *never* (1).
2. Physical activity scale: This scale included three items developed by the first and fourth researchers, showing how active the respondents were at night during Ramadan in the first 20 d. The respondents were asked to determine the degree to which they practised walking, going to gyms and playing sports with friends. A panel of experts from the College of Social Sciences (from sociology, social work and anthropology in biocultural, clinical and medical disciplines) assessed the items’ clarity, relevance and suitability for measuring physical activity at night during Ramadan in order to establish content validity, scored on a six-point Likert scale, ranging from *very much* (6) to *never* (1).

These two scales were created for this study. Construct validity was examined using exploratory factor analysis, which indicated that all items loaded onto a single factor, supporting the unidimensional acceptable of the scales. Tests and retests were performed to measure the stability of the instrument. Alpha Cronbach’s was used for reliability. The reliability was 0·84 and 0·70 for the two scales, respectively.

### Statistical analysis

SPSS (version 25) was used for data analysis. Descriptive and inferential statistical analyses were conducted. Percentages, means, sd and rankings were used to examine the extent to which different types of food were eaten at night. ANOVA was used to examine the differences among age groups and number of days visiting *Diwaniyyah* in the types of food eaten at night and among age groups and educational level in all samples, regularly visiting *Diwaniyyah* and not visiting *Diwaniyyah* on the dietary habit scale and physical activity scale. A *t* test was used to examine the differences among age groups and educational levels regularly and among those who did not visit *Diwaniyyah*. To examine the relationship between some demographic variables and *Diwaniyyah* visiting days and time, and types of food eaten at night, Pearson’s correlation analysis was used to predict the most associated and predicted variables of dietary habits and physical activity.

## Results

A total of one thousand men were approached, with 736 (74 %) completing the questionnaire. Response rate was 75·8 %. Participants ranged in age from 18–82 years. All participants were aged 18–82 years, and their average age was 41·54 years (sd *=* 15·622). Respondents came from different and various subgroups of the Kuwaiti population and from all six governorates (22·8 % from the Capital, 22·8 % from Hawalli, 11·8 % from Ahmedi, 17·1 % from Farwaniyyah, 12·3 % from Jahra and 11·1 % from Mubarak Al-Kabeer). Of the respondents, 32·2 % had completed high school or below, 45·5 % were college graduates and 22·3 % were postgraduates. Only 1·87 % of the questionnaires were excluded because of incomplete answers. Response rate of the questionnaire was very high. The initial response rate was almost 74 %.

To understand the most eaten types of food at night in Ramadan (after Iftar), Table 1 shows the percentages and means (sd) of the extent of food consumption to determine the most consumed types of food at night during Ramadan (after Iftar).

**Table 1.** Percentages, means and sd of the extent to which these types of food are eaten at night

Types of diet	Very much	Much	Middle	Little	Very little	Never	M	sd	Rank
	%	%	%	%	%	%			
Sweets	4·1	40·7	21·8	17·2	12·1	4·1	3·52	1·15	10
Pastry	3·3	12·1	35·9	25·2	16·7	6·8	3·40	1·19	11
Meat	6·5	19·4	46·9	14·9	8·2	4·1	3·89	1·14	3
Chicken	7·7	18·2	47·5	15·1	7·3	4·2	3·91	1·14	2
Fried	4·8	12·7	40·7	21·2	14·5	6·1	3·54	1·20	9
Vegetables	6·5	19·2	35·9	23·7	9·7	4·9	3·74	1·20	6
Fruits	6·2	16·4	32·0	23·6	16·6	5·2	3·57	1·26	8
Barbecue	5·0	11·7	30·8	23·5	16·3	12·7	3·27	1·35	12
Juice	3·1	8·5	21·4	22·4	21·6	23·1	2·80	1·39	14
Traditional sweets	7·7	16·8	34·6	19·2	16·4	5·2	3·65	1·29	7
Seafood	6·1	10·5	23·7	21·5	21·1	17·1	3·08	1·45	13
Dairy products	8·0	22·5	37·4	17·5	9·7	4·9	3·87	1·24	4
Whole grains	5·8	18·8	40·2	19·2	11·7	4·2	3·75	1·19	5
Rice	19·6	26·4	32·2	11·0	7·6	3·2	4·30	1·29	1

The results show that the food most eaten at night after Iftar in general was rice, chicken, meat and dairy products, with a high proportion of these types of food (Table 1). On the other hand, the less eaten types of food at night were juices, seafood (in general), barbeque and pastries, with a lower mean than the most popular eaten types of food (Table 1).

Table 2 compares these types of food eaten at night (after Iftar) by age group and the frequency of visiting *Diwaniyyah* by providing the mean, sd and *F*-values.

**Table 2.** Means, sd, *F*-ratios and *F*-values by age group for types of food eaten at night using one-way ANOVA

Age groups	20 years or below		21–40 years		41–59 years		60 years or above		*F*-value
	Mean	sd	Mean	sd	Mean	sd	Mean	sd	
Sweets	3·81	1·02	3·63	1·23	3·36	1·09	3·48	1·01	4·271**
Pastry	3·81	1·17	3·71	1·21	3·15	1·09	3·03	1·06	18·482***
Meat	4·28	1·27	4·17	1·11	3·75	0·989	3·35	1·02	20·810***
Chicken	4·08	1·29	4·20	1·11	3·80	1·02	3·42	1·26	15·959***
Fried	3·78	1·26	3·82	1·23	3·36	1·10	3·12	1·01	13·788***
Vegetables	3·12	1·38	3·67	1·16	3·92	1·14	4·11	1·03	13·032***
Fruits	3·28	1·23	3·47	1·13	3·61	1·15	3·99	1·91	6·479***
Barbecue	3·09	1·48	3·34	1·44	3·29	1·45	3·26	1·22	0·704
Juice	3·41	1·51	3·05	1·38	2·64	1·30	2·15	1·22	18·600***
Traditional sweets	3·99	1·33	3·83	1·30	3·51	1·21	3·37	120	6·592***
Seafood	2·58	1·61	3·06	1·43	3·14	1·43	3·33	1·27	8·847**
Dairy products	4·04	1·41	3·97	1·25	3·80	1·17	3·75	1·08	1·794
Whole grains	3·85	1·28	4·02	1·21	3·59	1·08	3·50	1·01	8·842***
Rice	5·04	1·24	4·59	1·20	4·07	1·21	3·74	1·12	26·312***
Late dinner (*Qabka*)	3·61	1·39	3·53	1·23	3·09	1·20	2·88	1·09	12·084***

**P < 0·01; ***P < 0·001.

Differences by age groups: The data in Table 2 show that there were significant differences among the four age groups in all types of food eaten at night except barbeque and dairy products. Younger age groups eat more sweets, pastry, meat, juices, traditional sweets, dairy products and rice than the other age groups (Table 2), and more generally, the younger age groups consume more of these types of foods at night during Ramadan. On the other hand, the older age groups had a higher mean for consuming vegetables, fruits and seafood at night during Ramadan. The older age group consumed more vegetables, fruit and other foods. For the middle-aged group, data show that they consume more chicken, fried food, barbeque and grains (bread and wheat) than the other age groups. They were also more likely to eat late at night (*Qabka*) than the other age groups.

Differences by visiting *Diwaniyyah* frequency: When comparing the types of food consumed at night during Ramadan with the frequency of visiting *Diwaniyyah*, Table 3 shows that there are significant differences among the frequencies of men visiting *Diwaniyyah* weekly during Ramadan in ten of the fourteen types of food eaten at night. The data show that men who usually go to *Diwaniyyah* daily have a higher mean consumption of sweets, pastry, meat, chicken, fried foods, juice, traditional sweets, dairy products and rice. The data show that the higher the frequency of visiting *Diwaniyyah*, the greater the consumption of these types of food at night. In addition, the data show that the higher the frequency of visiting *Diwaniyyah*, the higher the frequency of eating late at night (*Qabka*). No significant differences were found among the frequencies of men visiting *Diwaniyyah* weekly during Ramadan in their consumption of vegetables, fruits, barbeque, seafood or grains.

**Table 3.** Means, sd, *F*-ratios and *F*-values by number of days visiting *Diwaniyyah* for types of food eaten at night using one-way ANOVA

Number of days visiting *Diwaniyyah*	6–7 d a week		3–5 d a week		1–2 d a week		Less or never visit		*F*-value
	Mean	sd	Mean	sd	Mean	sd	Mean	sd	
Sweets	3·65	1·25	3·62	1·06	3·54	1·14	3·32	1·08	3·302*
Pastry	3·62	1·20	3·48	1·12	3·34	1·21	3·22	1·16	4·028**
Meat	4·14	1·12	3·97	1·06	3·73	1·16	3·79	1·07	5·160**
Chicken	4·14	1·13	3·91	1·10	3·87	1·20	3·81	1·02	3·063*
Fried	3·75	1·20	3·73	1·20	3·39	1·12	3·32	1·15	6·922***
Vegetables	3·74	1·23	3·84	1·19	3·70	1·11	3·77	1·21	0·493
Fruits	3·52	1·26	3·77	1·20	3·45	1·22	3·58	1·29	2·38
Barbecue	3·32	1·46	3·30	1·36	3·13	1·30	3·42	1·23	1·44
Juice	3·15	1·45	2·92	1·42	2·65	1·29	2·50	0·096	8·05***
Traditional sweets	4·03	1·23	3·80	1·33	3·52	1·22	3·29	1·19	11·99***
Seafood	3·20	1·54	3·02	1·40	3·06	1·41	3·11	1·43	0·516
Dairy products	4·03	1·22	3·97	1·21	3·70	1·21	3·86	1·21	2·588*
Whole grains	3·94	1·19	3·69	1·19	3·77	1·13	3·71	1·12	1·83
Rice	4·73	1·22	4·37	1·20	3·99	1·29	4·19	1·24	11·50***
Late dinner (*Qabka*)	3·86	1·22	3·68	1·12	2·95	1·07	2·57	1·09	53·75***

*P < 0·05; **P < 0·01; ***P < 0·001.

To examine the significant differences by age group and educational level of those regularly and never visiting *Diwaniyyah* in their dietary habits and physical activities during the nights of Ramadan, and to determine whether there were significant differences between regularly visiting the Diwaniyyah individuals during Ramadan, Table 4 shows these differences using one-way ANOVA and *t* tests.

**Table 4.** Means, sd, *F*-ratios and *F*-values by age group and educational level overall and in those regularly visiting *Diwaniyyah* and not visiting *Diwaniyyah* for dietary habit scale and physical activity scale scores using one-way ANOVA and differences between those regularly and not visiting *Diwaniyyah* by *t* test

	Over all			Regularly visiting *Diwaniyyah*			Did not visit *Diwaniyyah*			
	M	sd	*F*-value	M	sd	*F*-value	M	sd	*F*-value	*t*-value
Age groups	Dietary habit scale									
20 years or younger	55·78	10·47		57·6	10·27		53	10·12		0·183
21–40 years	56·06	9·94	13·864***	59·06	9·55	4·449***	51·52	10·27	0·506	4·41***
41–59 years	52·09	8·97		52·67	12·13		51·75	7·95		0·502
60 years or older	50·48	9·05		53·43	8·84		49·91	6·88		0·11
Age groups	Physical activity scale									
20 years or younger	9·27	3·51		9·67	3·57		8·46	3·05		1·21
21–40 years	9·5	3·68	54·598***	10·15	3·84	9·80***	8·69	3·31	14·3***	2·31*
41–59 years	6·69	3·14		8·1	4·01		5·93	2·91		3·44**
60 years or older	5·74	2·31		5·62	2·25		5·28	1·94		0·563
Educational level	Dietary habit scale									
High school or below	53·51	11·19		57·51	8·78		53·51	11·19		1·63
Graduate	54·36	10·46	3·14*	57·57	11·33	2·48*	54·36	10·46	0·091	3·73***
Postgraduate	51·93	8·54		52·87	11·14		51·93	8·54		0·87
Educational level	Physical activity scale									
High school or below	7·16	3·32		3·3	0·429		2·94	0·393		0·583
Graduate	8·49	3·78	9·99***	4·13	0·425	4·76*	3·29	0·39	0·143	3·24**
Postgraduate	7·68	3·68		4·19	0·765		3·32	0·456		1·73

*P < 0·05; **P < 0·01; ***P < 0·001.

Differences by age groups: Table 4 shows that there were significant differences by age group between men regularly visiting or never visiting *Diwaniyyah* on both the diet habit and physical activity scales, such that the older age groups had lower mean scores on the diet habit and physical activity scales. Older men eat fewer types of food and have lower physical activity levels at night during Ramadan than other age groups. The middle-aged group had a higher mean in all samples, whether regularly or never visiting *Diwaniyyah*. They consume more of the selected types of food and engage in more physical activity than other age groups.

Differences by visiting *Diwaniyyah* frequency: When examining the significant differences between men who regularly visit *Diwaniyyah* and men who do not, the data show that there are significant differences between these two variables in the dietary habit scale in the middle-aged group only. Men in this age group who regularly visit *Diwaniyyah* consume more food than men who do not. No significant differences were observed among the other age groups. There are also significant differences in physical activity scale scores between men who regularly visit *Diwaniyyah* and men who do not in two age groups (21–40 and 41–59 years old), such that men in the middle-aged groups visiting *Diwaniyyah* regularly practised more physical activity than those in the younger and older age groups.

Differences by educational level: Regarding educational level, Table 4 shows that postgraduate respondents showed a lower mean score on the dietary habit scale overall and in men regularly visiting *Diwaniyyah*. Men with higher education consume less food at night overall and in both those regularly or never visiting *Diwaniyyah* in Ramadan. Lower educational levels were associated with less physical activity than other educational levels. When comparing the educational levels of men who regularly visit *Diwaniyyah* and men who do not during Ramadan, the data show that the only significant difference is in those with a medium educational level (graduates). Men with graduate educational levels who regularly visit *Diwaniyyah* consume more types of food at night than those who do not visit the *Diwaniyyah*.

Table 5 presents the results of an examination of whether there were significant correlations of demographic variables (age, educational level and socioeconomic status) with the number of days visiting *Diwaniyyah* weekly and average hours spent there on weekdays and weekends, and with types of food eaten at night during Ramadan.

**Table 5.** Correlation coefficients between demographic variables, days visiting *Diwaniyyah* and time and types of food eaten at night

Variables	Age	Edu level	Seriocomic status
Diwaniyyah visiting			
No. days visit *Diwaniyyah*	*–*0·226**	−0·10*	0·070
Hours spend weekdays	−0·260**	−0·145**	0·001
Hours spend weekend	−0·345**	−0·173**	−0·021
Types of food			
Sweets	−0·117**	0·050	−0·012
Pastry	−0·262**	−0·022	−0·023
Meat	−0·289**	−0·041	−0·005
Chicken	−0·242**	−0·021	0·006
Fried	−0·222**	−0·039	0·013
Vegetables	0·218**	0·051	0·108**
Fruits	0·131**	0·063	0·129**
Barbecue	0·003	−0·014	0·046
Juice	−0·260**	−0·111**	−0·063
Traditional sweets	−0·186**	0·006	0·020
Seafood	0·122**	−0·026	0·109**
Diary	−0·095**	−0·007	−0·020
Whole grains	−0·155**	0·049	0·031
Rice	−0·326**	−0·121	−0·069
Late dinner (Qabka)	−0·234**	−0·020	0·076*

*P < 0.05; **P < 0·01.

Correlation by age and educational level: Significant negative correlations were found between respondents’ age and educational level, and both the number of days visiting *Diwaniyyah* weekly and the average hours spent there on weekdays and weekends, such that the higher the average number of days and hours on weekdays and weekends spent at *Diwaniyyah*, the younger the age group. In contrast, men with higher educational levels spend fewer days and hours on weekdays and weekends in *Diwaniyyah*. No significant correlations were found between socioeconomic status and the number of days and hours spent on weekdays or weekends in *Diwaniyyah*.

Correlation by demographic variables: For the correlation between the demographic variables and types of food eaten at night in Ramadan, Table 5 shows that there are negative significant correlations between age and the consumption of sweets, pastry, meat, chicken, fried, juice, traditional sweets, dairy, grains and rice at night during Ramadan, such that the younger the age, the greater the consumption of these types of food at night during Ramadan. Significant positive correlations were found between age and eating vegetables, fruits and seafood, meaning that the older the person, the more they eat those types of food. No significant correlation was found between age and the consumption of barbeque meals. Regarding participants’ educational levels, only significant negative correlations were found between educational levels and juice consumption. No significant correlations were found for other types of foods. Significant positive correlations were found between socioeconomic status and vegetable, fruit, and seafood consumption.

To predict the variables and factors that affect dietary habits and physical activity at night during Ramadan, Table 6 shows the association between dietary habit and physical activity scale scores with various variables using linear regression.

**Table 6.** Regression coefficients of social factors with dietary habit scale and physical activity scale scores

Variables	*B*	*β*	*t*-value
Dietary habit scale			
Age	−0·173	−0·270	−5·53***
Social individual	1·82	0·175	3·69***
No. days visiting *Diwaniyyah* weekly	0·437	0·105	2·07*
Adjusted *R* ^2^		0·105	*F =* 7·920***
Multiple *R*		0·120	
Physical activity scale			
Age	−0·095	−0·411	−9·14***
Economic status	0·412	0·221	5·16***
Social individual	0·535	0·142	3·25**
Adjusted *R* ^2^		0·241	*F =* 19·742***
Multiple *R*		0·254	

*P < 0·05; **P < 0·01; ***P < 0·001.

Association and prediction: Table 6 shows that age, social individuals and number of days visiting *Diwaniyyah* were associated with and predicted the number of types of food eaten at night during Ramadan. The data showed that age, socioeconomic status and social status were associated with and predicted physical activity.

## Discussion

The study findings reveal that lifestyle behaviour among Kuwaiti men change during Ramadan, including the timing and number of meals, the type and portion sizes of food consumed and patterns of physical activity. Within the ecological model of food and nutrition, individual diet is positioned at the centre of the model and refers to ‘the actual foods that individuals or groups consume to meet their nutrient needs’^(34)^. The findings illustrate how multiple layers of influence shape Kuwaiti men’s dietary behaviours during Ramadan. At the individual level, men largely maintain their usual diet but make minor adjustments to accommodate fasting requirements, including changes in meal timing, portion size, and the selection of foods that promote hydration and satiety. Age and educational level further contribute to variations in men’s daily routines and food choices.

At the interpersonal and social–environmental level, shared family meals and community gatherings during Ramadan reinforce social norms surrounding traditional Iftar dishes such as rice, meat and chicken, while limiting the consumption of foods like seafood, barbeque and pastries at night due to shared beliefs about comfort and hydration during fasting. At the cultural level, Ramadan strongly shapes food beliefs and traditions, with men favouring familiar dishes that reflect religious expectations and cultural heritage. Cross-cultural findings from Ghana and Bangladesh indicate that dietary changes during Ramadan vary across societies, highlighting the role of local cultural contexts^(21,35)^. At the socioeconomic and environmental level, food availability and income further influence dietary patterns^(36)^. In Kuwait, access to large traditional Iftar meals and the higher consumption of energy-dense foods among wealthier individuals exemplify how socioeconomic conditions shape Ramadan eating behaviours.

The study also revealed age-related differences in dietary intake during Ramadan, consistent with the ecological model of food and nutrition. At the individual level, younger men consumed more unhealthy foods, such as sweets, pastries and sugary dairy products, while older men chose more balanced, nutrient-rich meals^(37–39)^, likely reflecting greater health awareness and a higher prevalence of chronic conditions. At the interpersonal level, younger men appear more influenced by peer-oriented gatherings where high-carbohydrate foods are common, while older men were supported by family environments that encourage healthier eating. Culturally, although Ramadan emphasises celebratory sweets, younger men tended to engage more with these traditions, whereas older men prioritise health considerations. At the socioeconomic and environmental level, older adults often have more stable routines and better access to healthy foods, while younger men relied more on convenient, high-energy options available at night.

This study also found a positive association between Kuwaiti men’s night-time consumption of sugar- and carbohydrate-rich foods during Ramadan and their frequency of *Diwaniyyah* attendance. Men who attend *Diwaniyyah* daily – often after Taraweeh prayer and visiting multiple gatherings in one night – tend to consume traditional sweets, dairy products and pastries in line with cultural expectations of hospitality. These findings are consistent with previous studies reporting the negative health impacts of *Diwaniyyah* attendance, where large meals followed by sweets, coffee and tea serve as customary symbols of generosity^(2)^. Within the ecological model, the *Diwaniyyah* represents a key social-environmental influence, where shared foods carry cultural meaning, reinforce social bonds and shape men’s dietary practices during Ramadan.

In conclusion, this study found a significant effect of attending *Diwaniyyah* during Ramadan on Kuwaiti men’s lifestyle behaviours, including the type, amount and timing of food consumption, which might negatively affect their physical health, especially among younger respondents. Further longitudinal studies are suggested to examine the influence of attending *Diwaniyyah* during Ramadan among Kuwaiti men and whether their dietary habits change after the month ends or remain the same. In addition, comparative studies should be conducted to investigate the differences between men’s lifestyle behaviours and daily schedules of those attending and not attending *Diwaniyyah* during Ramadan. Since the Kuwaiti population is reported to have one of the highest rates of diagnosed cases of chronic disease, these studies are essential to assess men’s current health behaviour in order to predict their health outcomes in the future and acknowledge them through their social network, health centres and social media tools by reporting information about the impact of attending *Diwaniyyah* on men’s physical health.

### Limitation of the study

There are several limitations that should be acknowledged. One of them is employing an electronic questionnaire and a non-random sampling technique to gather data. Furthermore, recollection and social desirability biases could affect the use of self-reported data. Lastly, the results might not be very generalisable beyond the particular features of the research sample.
